# Plasma Processing Conditions Substantially Influence Circulating microRNA Biomarker Levels

**DOI:** 10.1371/journal.pone.0064795

**Published:** 2013-06-07

**Authors:** Heather H. Cheng, Hye Son Yi, Yeonju Kim, Evan M. Kroh, Jason W. Chien, Keith D. Eaton, Marc T. Goodman, Jonathan F. Tait, Muneesh Tewari, Colin C. Pritchard

**Affiliations:** 1 Clinical Research Division, Fred Hutchinson Cancer Research Center, Seattle, Washington, United States of America; 2 Department of Medicine, University of Washington, Seattle, Washington, United States of America; 3 Department of Laboratory Medicine, University of Washington, Seattle, Washington, United States of America; 4 Epidemiology Program, University of Hawaii, Honolulu, Hawaii, United States of America; 5 Human Biology Division, Fred Hutchinson Cancer Research Center, Seattle, Washington, United States of America; 6 Samuel Oschin Comprehensive Cancer Institute, Cedars-Sinai Medical Center, Los Angeles, California, United States of America; 7 Public Health Sciences Division, Fred Hutchinson Cancer Research Center, Seattle, Washington, United States of America; Innsbruck Medical University, Austria

## Abstract

Circulating, cell-free microRNAs (miRNAs) are promising candidate biomarkers, but optimal conditions for processing blood specimens for miRNA measurement remain to be established. Our previous work showed that the majority of plasma miRNAs are likely blood cell-derived. In the course of profiling lung cancer cases versus healthy controls, we observed a broad increase in circulating miRNA levels in cases compared to controls and that higher miRNA expression correlated with higher platelet and particle counts. We therefore hypothesized that the quantity of residual platelets and microparticles remaining after plasma processing might impact miRNA measurements. To systematically investigate this, we subjected matched plasma from healthy individuals to stepwise processing with differential centrifugation and 0.22 µm filtration and performed miRNA profiling. We found a major effect on circulating miRNAs, with the majority (72%) of detectable miRNAs substantially affected by processing alone. Specifically, 10% of miRNAs showed 4–30x variation, 46% showed 30-1,000x variation, and 15% showed >1,000x variation in expression solely from processing. This was predominantly due to platelet contamination, which persisted despite using standard laboratory protocols. Importantly, we show that platelet contamination in archived samples could largely be eliminated by additional centrifugation, even in frozen samples stored for six years. To minimize confounding effects in microRNA biomarker studies, additional steps to limit platelet contamination for circulating miRNA biomarker studies are necessary. We provide specific practical recommendations to help minimize confounding variation attributable to plasma processing and platelet contamination.

## Introduction

Circulating miRNAs were identified in human plasma and serum in 2008 [1]–[3]. Since then, considerable effort has been directed to the study of circulating miRNAs as biomarkers of diseases, including cancer, cardiovascular, obstetric and rheumatologic conditions [4], [5]. Despite excitement about the potential of miRNAs in disease prediction, prognosis and diagnosis, a variety of pre-analytical and analytical considerations need to be addressed to ensure valid scientific inference [6]. These include the establishment of standardized acquisition, processing and storage procedures, as well as the development of assays that are accurate, precise, specific and robust with regard to quantitation of miRNAs. There is growing recognition that pre-analytic variables such as differences in sample processing and handling can be sources of considerable variation in multiplex assays [7]. For example, plasma and serum processing [8], [9], choice of anti-coagulant [10] and hemolysis [8], [11] have been reported to affect miRNA measurement. Our work, and that of others, has shown strong correlations between whole blood cell counts and blood cell-derived plasma miRNAs, suggesting that baseline blood counts impact circulating miRNA measurement [9], [11]. In this study, we investigate the role of processing alone on circulating miRNA measurement.

We performed miRNA profiling of plasma from lung cancer cases and controls to discover potential circulating miRNA biomarkers for lung cancer early detection. Surprisingly, we found a global increase in abundance of most miRNAs in cases compared to controls, suggesting the presence of a systematic bias. One explanation was that a significant subset of circulating miRNAs originated from residual contaminating blood cells, since platelets and other blood cells are known to contain miRNAs [11]–[13]. Plasma is the cell-free fluid portion of blood and contains residual platelets and cellular debris, the amount of which varies with differences in processing. Plasma also contains microparticles, which are phospholipid microvesicles 0.05–1.5 µm in size and are released by platelets, blood cells and endothelial cells, and can persist in plasma and cellular blood products [14]. We hypothesized that differences in specimen processing affected the degree of residual cell and microparticle contamination and, in turn, the results of the lung cancer biomarker study, and could likewise be relevant to other intra- and inter-study comparisons.

To systematically investigate this, we evaluated the effects of stepwise processing on plasma specimens by using miRNA profiling to simultaneously evaluate the expression levels of 282 detectable miRNAs. We found that processing differences resulted in variation in residual platelet contamination in plasma and significant differences in miRNA abundance. We next examined whether quality control and processing steps after thawing from archival storage could help identify and eliminate the effects of pre-storage processing variability. Since serum is also a blood specimen type used for circulating biomarker studies, we used filtration to determine the extent to which platelets and microparticles affect serum miRNA abundance. Based on our results, we offer practical recommendations for blood specimen handling to optimize the reproducibility and interpretability of miRNA biomarker studies.

## Materials and Methods

### Ethics Statement

All specimens were obtained in accordance with the declaration of Helsinki guidelines and with ethics approval from the Institutional Review Boards at the Fred Hutchinson Cancer Research Center (FHCRC), University of Washington (UW) and University of Hawaii (UH).

After providing written informed consent, blood samples were obtained by standard phlebotomy using a 20-gauge butterfly needle from the antecubital vein. Subsequent procedures varied slightly between the different sites, and additional details follow.

### Standard Plasma Collected from 12 Lung Cancer Cases and 24 Healthy Controls (FHCRC)

Plasma from 12 non-small cell lung cancer cases receiving treatment and 24 healthy controls from a high-risk screening cohort, all at the Fred Hutchinson Cancer Research Center, were matched for age, ethnicity and smoking status for this experiment.

Cases and controls were processed using similar standard operating protocols for plasma preparation, with differences as detailed next: One 4 mL lavender top K_2_EDTA plasma tube (cases) or one each 10 mL and 4 mL lavender top K_2_EDTA plasma tubes (controls) were collected at each lab draw**.** Whole blood content was transferred to a 15 mL conical tube (cases) or was pooled from two tubes and divided equally into two 15 mL conical tubes (controls). Samples were immediately centrifuged at 1200*xg* at 4°C in either a Hettich Rotanta 460R benchtop centrifuge for 10 minutes with brake of 9 (cases) or an Eppendorf 5810R benchtop centrifuge for 15 minutes with brake of 2 followed by transfer to a fresh 15 mL conical tube and a second centrifugation at 1200*xg* at 4°C for 5 minutes with brake of 9 (controls). Plasma was removed, divided into 0.7 mL aliquots and immediately stored at −80°C. All downstream processing of frozen plasma of case and control sets were conducted simultaneously after interdigitating cases and controls to minimize batch effect.

### Stepwise Processing of Plasma and Serum from 3 Healthy Donors (UW)

The first 5 mL of blood were drawn into a discard tube during phlebotomy. One 10 mL red top serum tube labeled Tube #1 (BD Vacutainer 366430) and three 10 mL lavender top K_2_EDTA plasma tubes labeled Tubes #2–4 (BD Vacutainer 366643) were collected at each draw. From each draw we created a series of 7 differentially processed samples: platelet concentrate (Platelet_CONC_), platelet rich plasma (Plasma_RICH_), standard plasma (Plasma_STD_), platelet poor plasma (Plasma_POOR_), 0.22 µm filtered platelet poor plasma (Plasma_FILT_), standard serum (Serum_STD_) and 0.22 µm filtered serum (Serum_FILT_).

#### Standard serum and standard plasma

Tube #1 was allowed to clot at 25°C for 30 minutes. Serum Tube #1 and Plasma Tube #2 were centrifuged in an Allegra X-22 swinging bucket centrifuge (rotor SX4250) for 3400*xg* (4200 RPM) for 10 minutes at room temperature with high brake, as per standard University of Washington clinical lab protocol. The top three-fourths liquid volume of each tube was carefully transferred to a fresh 15 mL conical tube (leaving at least a 5 mm layer of serum or plasma behind to avoid disturbing the RBC/clot mass or buffy coat interface), and labeled accordingly.

#### Platelet poor plasma (PlasmaPOOR)

Remaining Plasma_STD_ was centrifuged at 1940*xg* (3000 RPM) at 25°C for 10 minutes in a Sorvall Legend RT centrifuge with no brake. The top three-fourths of supernatant was carefully transferred to a fresh tube and designated Plasma_POOR_.

#### Platelet rich plasma (PlasmaRICH)

Plasma Tubes #3 and #4 were centrifuged at 600*xg* at 25°C for 10 minutes in a Sorvall Legend RT centrifuge with no brake. Three-fourths of the plasma volume was carefully transferred into a fresh 15 mL conical tube and designated Plasma_RICH_.

#### Platelet concentrate (PlateletCONC)

After Plasma_STD_ was centrifuged and Plasma_POOR_ supernatant was removed as detailed above, the remaining pellet was gently resuspended in approximately 50 µl of residual liquid, avoiding introduction of bubbles or activation of platelets, and designated Platelet_CONC_.

#### Filtered serum (SerumFILT) and filtered platelet poor plasma (PlasmaFILT)

Aliquots of Serum_STD_ and Plasma_POOR_ were filtered through a 0.22 µm filter (Millipore Millex) and resulting filtrate was designated Serum_FILT_ and Plasma_FILT_, respectively.

### Standard Plasma and Serum Collected from 6 Healthy Donors and Post-archival Centrifugation (UH)

#### Study Design and Subjects

Blood specimens were collected as part of a longitudinal study of cervical human papillomavirus infection [15]. Briefly, healthy women, 18 years of age and older, were recruited from the University of Hawaii Student Health Service between 1999 and 2010 to participate. Study visits were scheduled at 4-month intervals. Blood samples were obtained at baseline and subsequent visits. For these experiments, we randomly selected serum and plasma (EDTA) samples collected from six Caucasian women at five different time points.

One 10 mL red top serum tube and one 10 mL lavender top K_2_EDTA plasma tube were collected at each lab draw. Blood samples were stored either refrigerated or on ice until processing within 2 hours of draw. Vacutainer tubes were centrifuged in a refrigerated Beckman Coulter Allegra X-12 centrifuge at 1825*xg* (2800 rpm) for 15 minutes with no brake. ***Serum_STD_.*** After clotting, serum was transferred from the red top vacutainer into a mixing tube, covered with Parafilm, and inverted 10 times. One mL was aliquoted into each of four 1-mL cryovials and stored at −70°C. ***Plasma_STD_***. Plasma was transferred from the lavender top vacutainer into a mixing tube, covered with Parafilm, and inverted 10 times. One mL of plasma was aliquoted into each of three 1-mL cryovials and stored at −70°C. ***Plasma_POOR_.***

(Performed at UW) After thawing, plasma was centrifuged at 1940*xg* (3000 RPM) at 25°C for 10 minutes in a Sorvall Legend RT centrifuge with no brake. The top three-fourths of supernatant was carefully transferred and designated Plasma_POOR_.

### Measurement of Complete Blood Counts

The complete blood count with differential was measured from all samples on the Sysmex Automated Hematology Analyzer at the University of Washington Clinical Laboratory.

### Particle Measurements

Particle counts were measured on a Beckman Multisizer™ 4 Coulter Counter®, which quantifies particles in suspension by measuring changes in electrical conductivity as particles pass through an aperture of 20 µm and allows accurate measurement of particles in the 0.4–10.0 µm size range. Following manufacturer’s protocol, samples were mixed with filtered Isoton® II diluent, inverted 2–3 times immediately prior to measurement and analyzed with the Coulter Counter®. The number of particles per µL in the size-range of platelets (1.5–3.0 µm) and microparticles (0.4–1.5 µm) were calculated using the Coulter Counter® gating software.

### RNA Isolation

RNA isolation was performed in duplicate for each specimen as previously described [11]. Briefly, the miRNeasy Kit (Qiagen) was used, following the manufacturer’s protocol with the following modifications: For each sample replicate, 200 µl was mixed with 5 sample volumes (1 mL) of QIAzol® reagent and immediately vortexed for 15 seconds. After a 5 minute incubation at 25°C, samples were stored at −80°C. Frozen samples were thawed on ice, 13.5 µL of a spike-in mixture containing synthetic *Caenorhabditis elegans* miRNA oligonucleotides cel-miR-39, cel-miR-54 and cel-miR-238 (each at 30 fmoles/µL) prepared in QIAzol® were added to each sample, vortexed, and 0.2 volumes of chloroform added. The manufacturer’s protocol was followed thereafter with each sample replicate loaded individually onto an affinity column, eluted in 50 µl and promptly frozen/stored at −80°C.

### microRNA Profiling using qRT-PCR Array

Plasma and serum RNA were profiled for the relative abundance of 365 miRNAs by miRNA Ready-to-Use PCR, Human panel I, V1.M qRT-PCR arrays (Exiqon), as previously described [11]. Fourteen experimental arrays were applied to profile two biological replicates for each of 7 sample types (Platelet_CONC_, Plasma_RICH_, Plasma_STD_, Plasma_POOR_, Plasma_FILT_, Serum_STD_ and Serum_FILT_). qPCR was performed on a Viia™7 instrument (Applied Biosystems), analysis was done using the Viia™7 software (V1.0), and cycle threshold (CT) values were calculated using manual constant thresholding. miRNA assays were normalized between the 14 plates using the UniSp6 and Inter-Plate Calibrator control assays, as previously described [11], including the designation of “not detected” (ND) to samples with CT values above the limit of detection, or if the average CT for the individual miRNA assay was greater than the no template control (NTC) array. Assays were median normalized. For analyses between groups, the number of detectable miRNAs was defined as the number of assays reliably detected in at least 2 of the 7 processing conditions, which was 282.

### Individual miRNA TaqMan Quantitative Reverse Transcriptase PCR

Individual miRNAs were measured by qRT-PCR as previously described [16]. We used TaqMan® assays (Applied Biosystems) for human miRNAs hsa-let-7a, hsa-miR-16, hsa-miR-92a, hsa-miR-122, hsa-miR-124a, hsa-miR-141, hsa-142-3p, hsa-miR-205, hsa-miR-210, hsa-miR-223 and hsa-miR-451 and the *C.elegans* miRNAs cel-miR-39, cel-miR-54, and cel-miR-238.

### Statistical Analysis

The difference between platelet and microparticle content in lung cancer cases compared to controls and in archival plasma and serum was calculated using Student's t-test. The difference between expression of the individual miR-142-3p, let-7a, miR-223, miR-16, miR-451 and miR-122 across the sample types Platelet_CONC,_ Plasma_RICH_, Plasma_STD_, Plasma_POOR_ and Plasma_FILT_ was calculated with the cycle thresholds (CTs) using Student's t-test. To determine whether CTs of miRNA expression were significantly changed due to processing, Wilcoxon signed-rank test was performed. Correlation between miRNAs most affected by processing and those highly expressed in platelets was performed using a Spearman test. Fold changes described are calculated from the differences of 2^(ΔCT)^. To analyze variability between all groups by one-way ANOVA (multiclass response test), significance analysis of microarrays [17], including false discovery rate analysis, was conducted. We chose FDR<1% as a conservative cutoff, in part based on prior precedent FDR cutoffs in miRNA profiling studies [18]. All tests were two-sided, and *p*<0.05 was considered statistically significant. Analyses were conducted using GraphPad Prism (version 5c) and R package (version 2.0).

## Results

### Differences in Global miRNA Expression between Lung Cancer Cases and Controls are Associated with Differing Residual Platelet Contamination in Plasma Specimens from Cases and Controls

**Figure 1:**
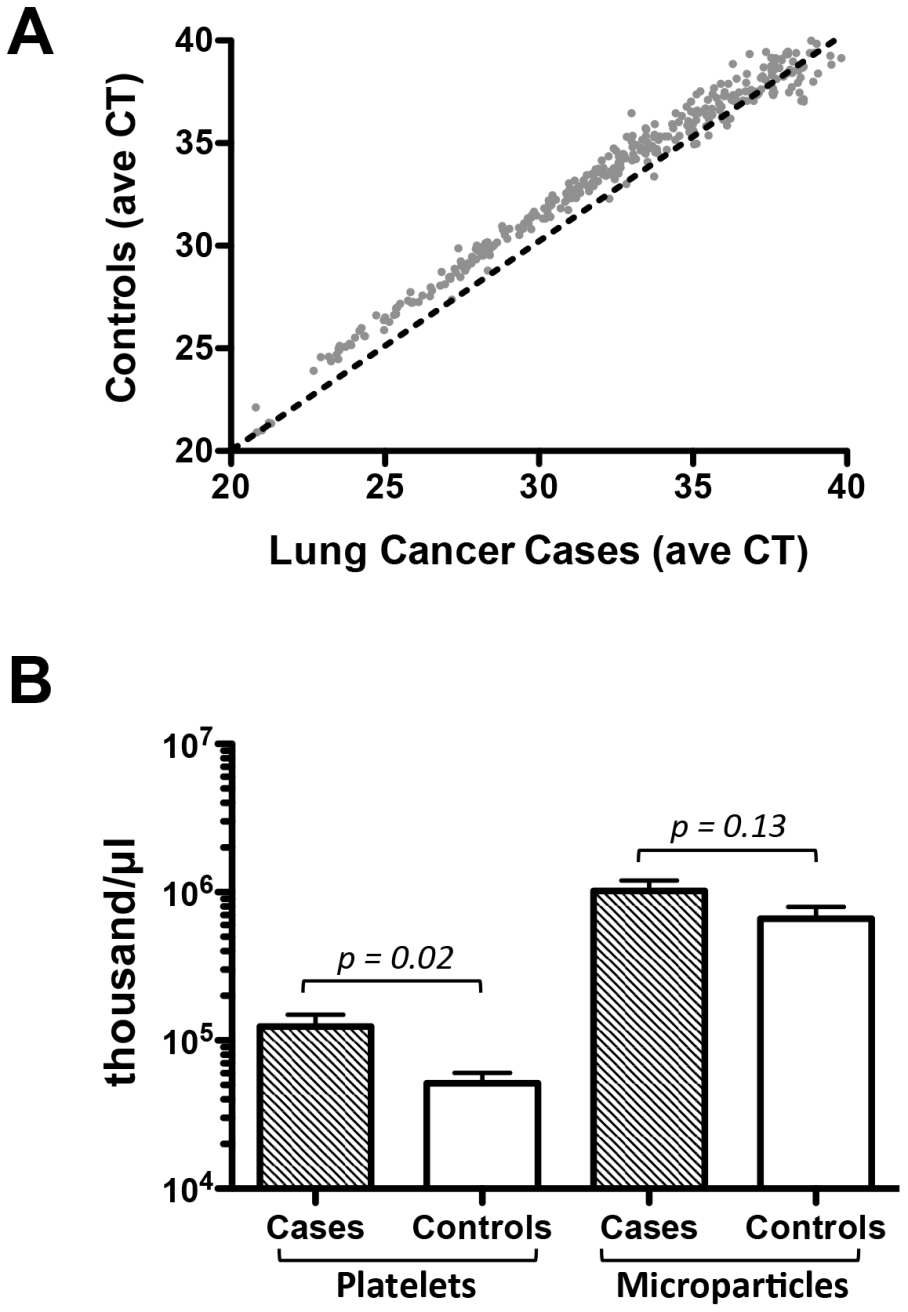
MicroRNA Biomarker Study Comparing Plasma from Lung Cancer Cases and Controls. *(A)* Average miRNA cycle threshold (CT) of plasma from lung cancer cases on the x-axis and from controls on the y-axis. Dotted line represents the line of identity. *(B)* Bar graph of average particle counts (thousand/µL) in the platelet and microparticle size ranges of plasma from lung cancer cases and controls. Student's t-test, two-tailed, *p = *0.02 and 0.13, respectively. Error bars represent standard deviation.

We participated in a study to identify candidate circulating miRNA biomarkers for early detection of lung cancer by performing miRNA profiling by qRT-PCR array on plasma specimens from 12 lung cancer cases and 24 matched healthy controls. The total number of miRNAs (including controls) detected in the cancer cohort was 356 and the total number detected in the control cohort was 348. Analysis of differential expression of circulating miRNAs showed a global increase in miRNA expression in cases compared to controls (**Figure 1A**), suggesting the presence of a systematic confounding factor.

To examine if the miRNA expression differences observed may have resulted in part from differences in platelet contamination, we measured the platelet and microparticle content of samples and found that platelet content of the lung cancer cases was on average 2.4x higher than of the control group (Student’s t-test, *p = *0.02, **Figure 1B**). The microparticle content was 1.6x higher in cases compared to controls, did not reach, but tended to statistical significance (Student’s t-test, *p = *0.13).

### Residual Platelet and Microparticle Content of Plasma is Affected by Blood Processing Steps

**Figure 2:**
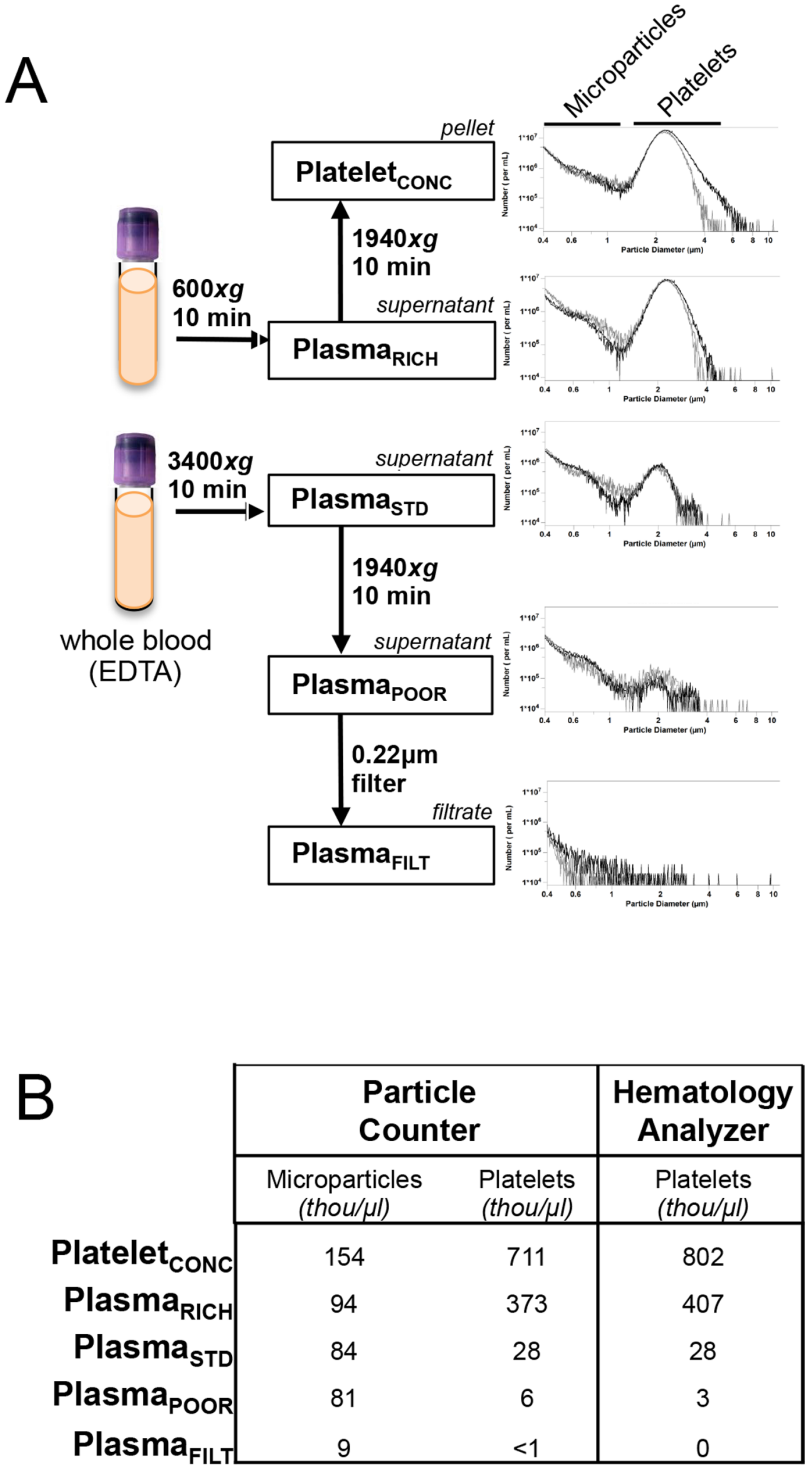
Stepwise Plasma Processing and Associated Particle Content. *(A) Left:* Schematic of stepwise plasma processing steps. *Right:* Histogram content of platelets and microparticles in stepwise processed plasma samples. X-axis is particle diameter and Y-axis is number of particles/mL. *(B)* Platelet and microparticle content (in thousand/µL) of stepwise processed plasma samples as measured by particle counter and hematology analyzer.

To systematically evaluate the effect of individual processing steps on platelet content and miRNA expression, we created differentially processed plasma samples (**Figure 2A**). (Serum results will be presented in a separate section).

Complete blood count measurements demonstrated that Plasma_STD_ contained an average of 28 thousand/µL residual platelets as measured by a hematology analyzer (**Figure 2B**). Automated hematology analyzers have poor precision at low platelet counts, so we also used a specialized particle counter to quantify particles in the size range of platelets (1.5–3.0 µm) and microparticles (0.4–1.5 µm) and found close concordance between platforms (**Figure 2B**).

Processing Plasma_STD_ into Plasma_POOR_ samples removed 80–90% of residual platelets (Figure 2B). Additional 0.22 µm filtration to produce Plasma_FILT_ resulted in removal of 99–100% of residual platelets relative to Plasma_STD_. Conversely, Plasma_RICH_ and Platelet_CONC_ had 13–19x and 25–30x increased platelet content compared to Plasma_STD_, respectively.

### Circulating MicroRNA Levels in Plasma are Significantly Altered by Differences in Blood Processing Steps

To determine the abundance of circulating miRNAs in the differentially processed samples, we profiled miRNA expression in the differently processed samples. The number of detected miRNAs (out of a possible 365) for each sample type was as follows: Platelet_CONC_: 315, Plasma_RICH_: 325, Plasma_STD_: 277, Plasma_POOR_: 279, Plasma_FILT_: 262, Serum_STD_: 274, Serum_FILT_: 271. Of the 365 miRNAs tested, 282 miRNAs were reliably detected in at least 2 of the 7 processing conditions and were used for downstream analysis. Seventy two percent (203/282) of detected miRNAs were affected by processing when looking across all 7 conditions (using a false discovery rate cutoff of <1%). (Serum_STD_ and Serum_FILT_ results will be presented in a separate section).

**Figure 3:**
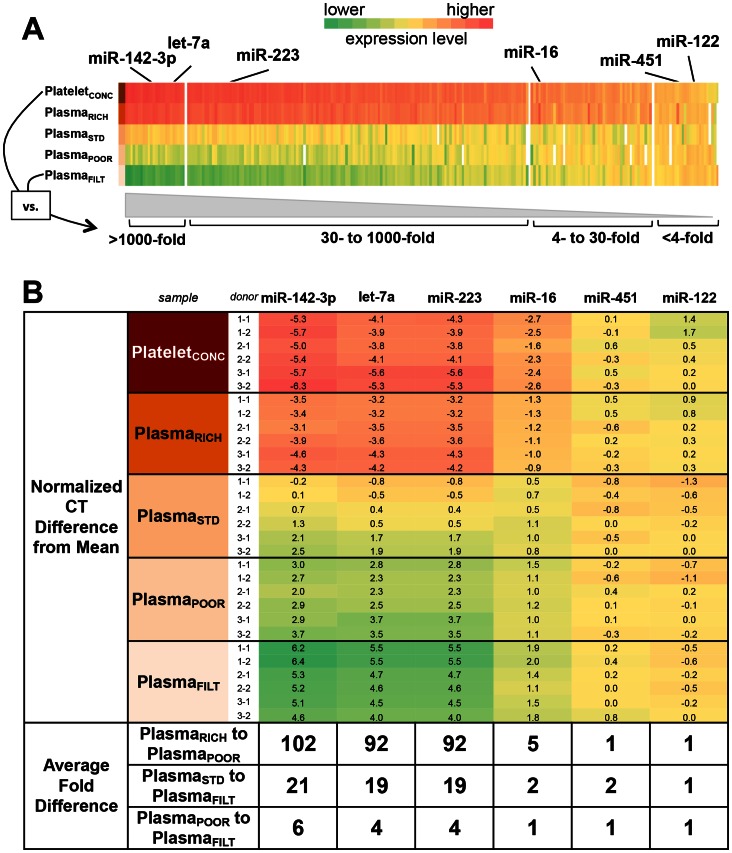
MicroRNA Expression in Stepwise Processed Plasma Samples Using qRT-PCR Profiling. *(A)* Heat map of relative expression (in CTs) of 282 detected miRNAs assayed in parallel using the Exiqon qRT-PCR array. Colors represent the greatest relative expression differences across the 5 different processing conditions for each individual miRNA. The processing condition(s) with the highest relative expression is shown in red and lowest in green for each miRNA. miRNAs are arranged left to right in order of decreasing difference between CTs across all 5 samples. Selected miRNAs are highlighted above. Ranges of average fold-differences are indicated along the bottom, where fold-differences are calculated as = 2^(ΔCT)^. *(B)* Validation of selected miRNAs by individual qRT-PCR assay. MicroRNAs are arranged in order of decreasing range between highest and lowest fold difference. Numbers in each box represent the normalized CT difference from the mean of all CT values for each miRNA evaluated in sample type. Color schema follows panel A. Average miRNA expression fold-differences between Plasma_RICH_ vs. Plasma_POOR_, Plasma_STD_ vs. Plasma_FILT_, and Plasma_ POOR_ vs. Plasma_FILT_ is indicated in the bottom rows. Fold-differences are calculated as = 2^(ΔCT)^.

Comparing the 5 differently processed plasma samples, 10% (27/282) of miRNAs were differentially expressed by 4–30x (ΔCT 2–5), 46% (131/282) by 30–1000x (ΔCT 5–10), and 15% (41/282) by ≥1000x (ΔCT ≥10) (**Figure 3A** **, Table S1**). For example, expression of proposed circulating cancer biomarker let-7a was altered as much as ∼1300x between the 5 plasma samples (Platelet_CONC_: 22.7±0.3 to Plasma_FILT_: 33.1±0.8; Student's t-test, *p*<10^−5^). A minority of miRNAs, 7% (21/282) were relatively resistant to processing, with <4-fold (ΔCT <2) differences (**Figure 3A**).

Six miRNAs representing the spectrum of most to least affected by processing were selected for validation using individual qRT-PCR assays and found to have good concordance (**Figure 3B**). Specifically, miR-142-3p, let-7a, miR-223, and miR-16 were highly affected while miR-451 and miR-122 were minimally affected by plasma processing.

The fold changes discussed above signify absolute changes in miRNA abundance, but we also wanted to know the effect on relative abundance (also known as rank order of expression). We found, for example, that miR-26a and miR-24 ranked in the top 10 expressing miRNAs in Plasma_STD_, but ranked as 31^st^ and 16^th^, respectively, in Plasma_FILT_ (**Table S1**). Moreover, rank orders of all miRNAs were significantly changed when comparing Plasma_STD_ with Plasma_FILT_ (Student's t-test, *p*<0.0001) and Platelet_CONC_ with Plasma_FILT_ (Student's t-test, *p*<0.0001) in the setting of an expected correlation of miRNAs between Plasma_STD_ and Plasma_FILT_ and between Platelet_CONC_ and Plasma_FILT_ (Spearman r = 0.87 and 0.76, respectively).

### The Subset of MicroRNAs Most Affected by Processing of Plasma Corresponds to the MicroRNAs with Highest Expression in Platelets

To determine if the miRNAs most affected by processing were also those with high expression in platelets, we performed a correlation analysis and found a correlation (Spearman r = 0.43, *p*<0.0001) between the miRNAs most affected by processing and those most highly expressed platelets [11].

### Addition of Processing Steps after Archival Storage can Effectively Reduce the Residual Platelet and Microparticle Content of Archived Plasma and Serum Samples

**Figure 4:**
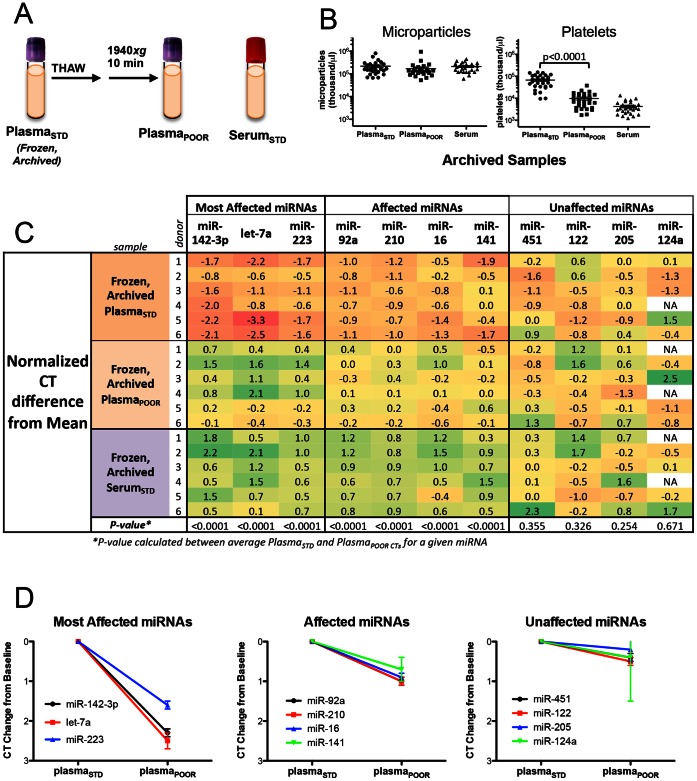
Additional Quality Control and Processing of Frozen, Archived Plasma Samples. *(A)* Schematic of archival sample types: Plasma_STD_, post-thaw processing to Plasma_POOR_, and Serum_STD_ Samples were collected at each of five time points from six healthy donors in a prospective screening cohort between 2001 and 2007. *(B)* Microparticle and platelet content of 3 sample types from all 30 timed draws as measured by particle counter. *(C)* Normalized CT difference from the mean of selected miRNAs tested by individual qRT-PCR assay. Individual miRNAs are grouped into most affected, affected and unaffected by processing, as listed. T-test comparing Plasma_STD_ and Plasma_POOR_ was calculated for each miRNA and p values are listed on the bottom row. *(D)* Graphs depicting the CT change from baseline of the miRNAs most affected, affected and unaffected in frozen archival Plasma_STD_ and Plasma_POOR_.

To address whether additional processing steps after thawing from archival storage could eliminate pre-storage processing variables, we used archived plasma and serum collected at 5 different time points, over a period of 6 years, from 6 healthy female donors. We compared frozen archived Plasma_STD_, frozen archived plasma with an additional centrifugation step after thaw (Plasma_POOR_), and frozen archived Serum_STD_ (**Figure 4A**).

We found that the microparticle content was similar in all three sample types (**Figure 4B**, left). However, the platelet content of Plasma_POOR_ was significantly reduced compared to Plasma_STD_ (Student’s t test, *p*<0.0001, **Figure 4B**, right), indicating the effectiveness of centrifugation in reducing platelet contamination in archived plasma.

We evaluated the six miRNAs representing the spectrum of most to least affected by processing and 5 additional candidate miRNA biomarkers by individual qRT-PCR. We also examined the correlation between expression of miRNAs and platelet contamination and found agreement with earlier findings (**Figure S1**). The pattern of miRNAs most, moderately, and least affected by additional centrifugation of archival plasma corresponded with our earlier findings in fresh plasma (**Figure 4C**).

### The Spectrum of Circulating MicroRNA Abundance in Serum is Moderately Altered by Filtration and Differs from Plasma

**Figure 5:**
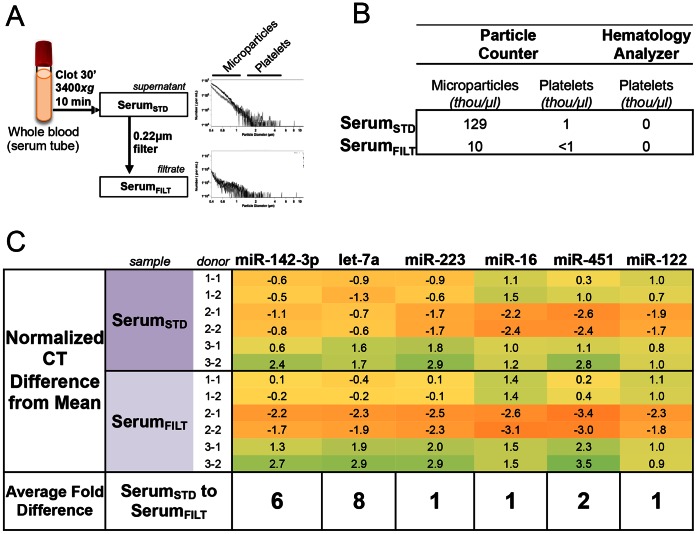
Serum Processing and Associated Particle Content. *(A) Left*: Schematic of Serum_STD_ processed with a 0.22 um filtration step to form Serum_FILT_. *Right:* Histogram content of platelets and microparticles in stepwise processed plasma samples. X-axis is particle diameter and Y-axis is number of particles/mL. (B) Platelet and microparticle content (in thousand/µL) of serum samples as measured by particle counter and hematology analyzer. *(B)* Validation of selected miRNAs by individual qRT-PCR assay. Normalized CT difference from mean of all 7 samples (including 5 plasma samples) is shown. MicroRNAs are arranged as in Figure 3. Average fold difference between Serum_STD_ vs. Serum_FILT_ is indicated below.

Since serum is also a sample type used for circulating biomarker studies, we compared Serum_STD_ with Serum_FILT_ to determine the effect of additional filtration (**Figure 5A**). Particle counting showed removal of microparticles by filtration, but platelet content did not differ dramatically (**Figure 5B**). miRNA profiling showed that 6% (16/282) of detectable miRNAs showed ≥4x (ΔCT ≥2) difference at a false discovery rate cutoff <1% (**Table S2**). Individual qRT-PCR of six miRNAs gave similar results (**Figure 5C**). Serum miR-142-3p and let-7a showed differences in expression of 6x and 8x, respectively, after filtration.

We also compared Serum_STD_ with Plasma_STD_ to define the differences in miRNA expression between the two sample types. We found that 6% (17/282) of microRNAs showed ≥4x difference in expression, indicating there are relatively few but important qualitative differences in miRNA expression of serum compared to plasma (**Table S3**).

## Discussion

Circulating miRNAs have received much attention as attractive candidates for non-invasive biomarkers for a variety of disease processes, but are not yet in clinical use. This may, in part, be due to under-recognition of the effects of sample processing and other pre-analytic variables on circulating miRNA measurement in early biomarker studies. We previously reported that a majority of circulating miRNAs are expressed in one or more blood cell types [11]. Here, we show that standard laboratory protocols for plasma sample processing result in significant differences in platelet content, and that residual platelet contamination is a significant confounding source of circulating miRNAs.

In our lung cancer biomarker study we found a global increased expression of most miRNAs as well as significant differences in platelet content (and, to a lesser degree, microparticles) in cancer cases compared to controls. This suggests that differences in processing conditions may have confounded miRNA measurement and hampered discovery of circulating miRNA biomarkers. It is possible that higher baseline platelet counts in lung cancer cases compared to controls may have also been a contributing factor as there is evidence that thrombocytosis is a poor prognostic feature of patients with lung cancer and other malignancies [19]–[21]. This further emphasizes the importance of controlling for platelet content in plasma samples to avoid incorrectly attributing differences in circulating miRNAs directly to a disease process, rather than to differences in residual platelet content. Regardless of the degree to which baseline platelet counts contribute to residual platelet content, our data clearly indicate that differences in processing can also be a major confounder to circulating miRNA studies and merits careful attention.

Results of earlier studies have highlighted the effect of differences in processing on selected panels of miRNAs, but have been relatively limited in scope [8], [9], [22]. Here, we used miRNA profiling to systematically examine stepwise sample processing steps, which allowed parallel examination of expression of 282 miRNA and a more accurate assessment of effect. We were surprised to find that the majority of miRNAs (72%) exhibited significant differences in expression attributable solely to differences in processing. Additional centrifugation and filtration steps added to standard plasma also resulted in dramatic differences, with over a third of miRNAs showing 4x to over 30x changes in expression. Many miRNA biomarker studies report differences that fall within these fold ranges. In addition, since biomarker discovery studies frequently consider relative (or rank order) expression, for example when cut-points are used, we also and found the rank order of miRNA expression was significantly altered as a result of processing.

Platelets and microparticles are decreased in a stepwise fashion after plasma processing steps. While the majority of effect on miRNA expression is due to residual platelet differences, we also observed differences in microparticles. Filtration differs somewhat from centrifugation in that it removes not only platelets/cell debris, but also removes microparticles to create a truly platelet-eliminated plasma (**Figure 1A**). The majority of effect that we observed came from platelet contamination, but a smaller effect was seen from microparticles, which is not surprising since microparticles are derived, in part, from platelets [14]. Importantly, some miRNAs, such as miR-122 are not impacted by either centrifugation or filtration, suggesting they are in fact independent of blood cell contamination. It should also be noted that we were only able to assess microparticles in the 0.4–3.0 µM size range. Smaller microparticles known to contain miRNAs, such as exosomes, are in the 10–100 nM range, and would not be expected to be removed either by the centrifugation or filtration conditions used in this study.

The correlation between the miRNAs most affected by processing and the miRNAs with highest expression in platelets strongly suggest that platelets and platelet-derived microparticles, in particular, are likely contributing to miRNAs in plasma. It is also important to note that although miR-142-3p, let-7a and miR-223 are highly expressed in platelets, platelets are not the only source of these miRNAs, and therefore the degree to which they are relatively affected by platelet contamination is not linear. Of concern, miR-142-3p, let-7a and miR-223, have been proposed as circulating cancer biomarkers [23]–[26]. Our analysis also suggests that for many miRNAs, plasma processing variability accounts for the majority of observed variance, in some cases more than biological variance (**Figure S2**). In addition, others have recently noted that platelet-derived miRNAs dominate the profiles of plasma samples [27], [28], consistent with our observations, and further emphasizing the importance of controlling for the confounding effects of platelet contamination.

Perhaps most importantly, we have identified a means to minimize the confounding effects of platelet contamination occurring after standard laboratory protocols and prior frozen archival storage. Our data indicate that additional quality control and processing steps after thawing from frozen archival storage can minimize the effects of processing variability occurring prior to archival storage. This is important since there is great interest in profiling miRNA in archived plasma samples from existing cohorts, where processing conditions may not have been optimized with miRNA measurement in mind, or may contain subtle differences such as in our lung cancer study. Using plasma and serum stored up to 6 years, we showed that measuring platelet content and adding a differential centrifugation step can effectively remove platelet contamination from archived samples, thus facilitating more controlled assessments of cell-free, circulating miRNAs. To our knowledge, this is the first description of post-storage quality control and processing measures for plasma miRNA.

There are a number of limitations to this study. Although we show evidence that heterogeneous processing conditions are critically important to consider due to their effects on miRNA measurement, we do not offer a standard optimized protocol for general applications. Similarly, we do not generically recommend serum over plasma as an analyte. Our study also does not rigorously investigate additional factors such as phlebotomy conditions, EDTA container types or the specific effects of centrifugation brake, temperature and speed conditions. Circulating miRNAs have a wide array of potential applications, and optimal processing will vary depending on the context of the specific miRNAs of interest and the questions asked.

The potential of circulating miRNAs for biomarkers of cancer and other diseases will be greatly enhanced if a number of practical recommendations are followed (**Box 1**). First, consider obtaining a complete blood count and/or using a particle counter to assess the degree and variability of platelet contamination in plasma specimens. Consider an additional centrifugation or filtration step to remove/equalize platelet and microparticle content between samples. Although not within the scope of this paper, we also recommend assessment of the degree of hemolysis in plasma and serum samples [8]. This is particularly important because hemolysis cannot be removed by centrifugation or filtration, but could be corrected and/or accounted for if miRNAs of interest are red cell associated, such as miR-451. Our results suggest that miRNAs should be preferentially selected for further study based on inherent resistance to processing effects if possible. Some miRNAs, such as miR-122 (one of the few tissue specific miRNAs), are relatively unaffected by the processing effects described here. Finally, we show that there are important differences between serum and plasma, and the decision of which sample type to use or collect should be weighed against the particular miRNAs of interest and the samples available. While serum has less platelet contamination at baseline than plasma, some miRNAs are differentially expressed between serum and plasma (**Table S2**). Therefore, the best specimen type is expected to vary based on the proposed application and miRNAs of interest.

Our recommendations are of particular importance and urgency given the increasing prevalence of multi-institutional translational biomarker studies and the increased likelihood of subtle and systematic processing differences within an experimental set. We are optimistic that greater attention to processing protocols and quality controls measures will help realize the promise of circulating miRNA biomarkers.

### Recommendations for Sample Processing and Quality Control in Circulating Biomarker miRNA Studies

- Perform platelet counts on plasma samples after processing
  1. Reject samples with platelet count above a predefined threshold.
- Consider addition of pre- or post-storage centrifugation or filtration
  1. A second centrifugation step will remove most of the residual platelets but not all. If a biomarker candidate is especially sensitive to platelet contamination, filtration may be best.
- Whenever possible, choose miRNAs that are less affected by processing
  1. Note the platelet and other blood cell expression levels of a biomarker candidate when interpreting results
- Measure hemolysis and its consider effects on miRNAs abundant in red blood cells.
- Consider differences in expression of miRNAs of interest in serum vs. plasma

## Supporting Information

Figure S1 **Correlation Between Expression of miRNAs and Platelet Content.** Each miRNA is represented as a separate graph. Graphs depict miRNA expression in CTs (Y-axis) as measured by qRT-PCR and quantity of platelet-sized (1.5–3.0 µm) particles as measured with by particle counter (X-axis). For each miRNA, the Pearson correlation coefficient r and p value are listed in the top right of each graph. MiRNAs most correlated with platelets are shown in the left column. MiRNAs somewhat correlated with platelets are shown in the center column. MiRNAs not significantly correlated with platelets are shown in the right column.
(TIF)

Figure S2 **Percentage Contribution of Total Variance in miRNA Expression from Processing and Analysis Steps in Archival Blood Specimens.** Contribution of total variance in miRNA expression from plasma processing, RNA preparation, qRT-PCR in the frozen archived plasma samples. Analysis of estimating components of variances to measure variation from pre-analytic (study subjects) and analytic factors (specimen type and RNA preparation) using the archived samples was conducted. This approach was based on the concept that total variance of the expression of miRNA was equal to the sum of the variances of the components [1], [2]. miR-205, miR-124a, miR-141, and miR-122 were excluded due to missing data (52%, 95%, 39%, and 16%, respectively). Among the remaining seven miRNAs, total variance ranged from 1.46 (miR-16) to 5.42 (miR-142-3p). Inter-assay variance contributed to most of the total variance (38%–85%). Intra-assay variance was ranged from 0.33 (miR-451) to 3.36 (miR-143-3p) among the seven miRNAs. Within intra-assay factors (centrifugation, RNA preparation, and duplicated PCR), centrifugation contributed more than 50% among eight of eleven miRNAs and the range was from 91.1% to 100.0%. (Not shown is the contribution of biological variance, which makes up the remaining difference.) 1. McDonald JS, Milosevic D, Reddi HV, Grebe SK, Algeciras-Schimnich A (2011) Analysis of circulating microRNA: preanalytical and analytical challenges. Clin Chem 57∶833–840. 2. Tichopad A, Kitchen R, Riedmaier I, Becker C, Stahlberg A, et al. (2009) Design and optimization of reverse-transcription quantitative PCR experiments. Clin Chem 55∶1816–1823.
(TIF)

Table S1 **Top 10 Expressing miRNAs in Standard Plasma with Corresponding Expression Rank in Differently Processed Plasma.**
(XLSX)

Table S2 **Table of Differentially Expressed miRNAs in Plasma vs Serum.**
(XLSX)

Table S3 **qRT-PCR Array Data of 7 Plasma and Serum Sample Types.**
(XLSX)
