# Supplementary figures and images for: Plasma Processing Conditions Substantially Influence Circulating microRNA Biomarker Levels
Source: PLoS One. 2013 Jun 7;8(6):e64795. doi: 10.1371/journal.pone.0064795 (PMC3676411; doi:10.1371/journal.pone.0064795)

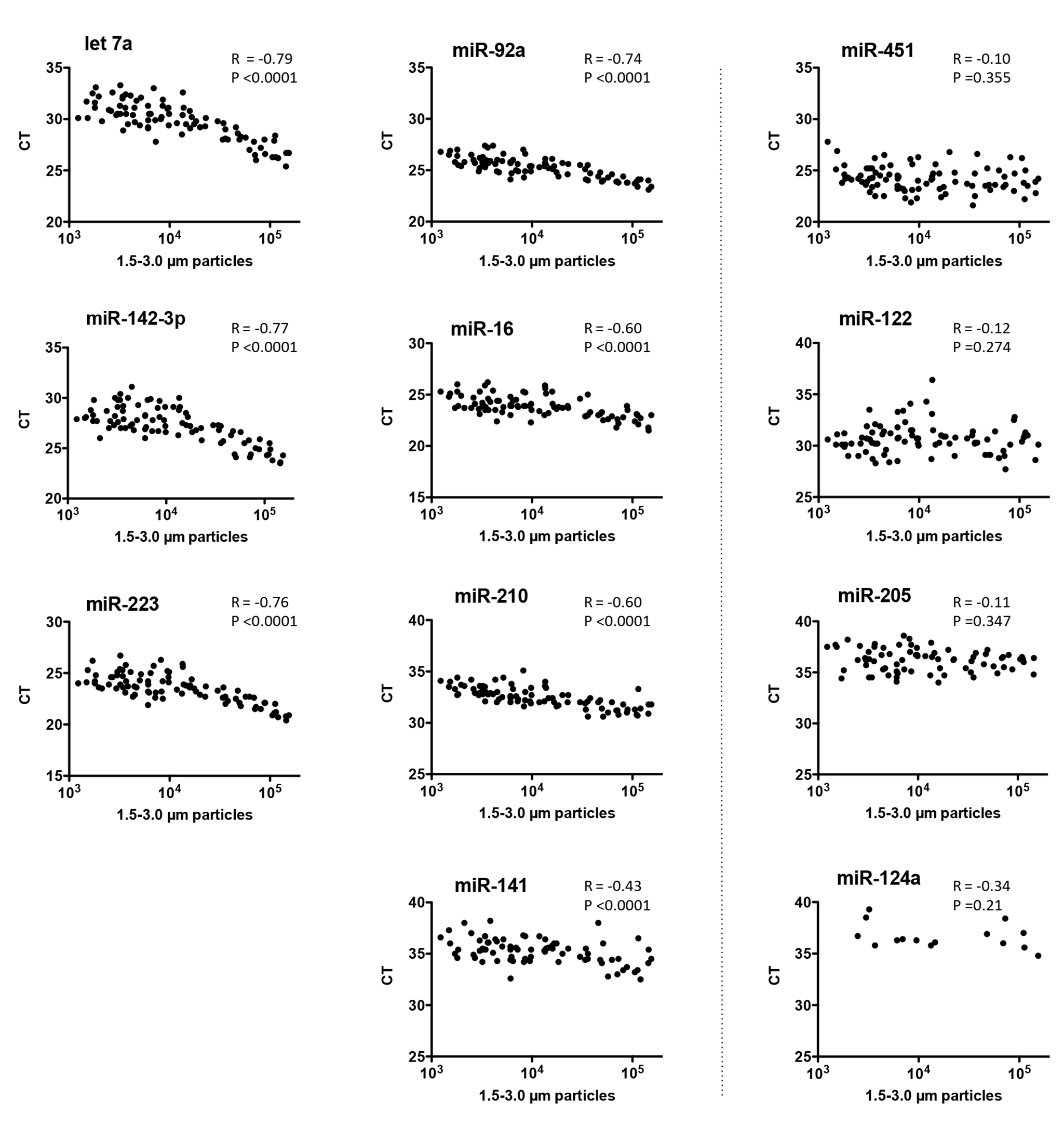

Supplement: Figure S1 — Correlation Between Expression of miRNAs and Platelet Content. Each miRNA is represented as a separate graph. Graphs depict miRNA expression in CTs (Y-axis) as measured by qRT-PCR and quantity of platelet-sized (1.5–3.0 µm) particles as measured with by particle counter (X-axis). For each miRNA, the Pearson correlation coefficient r and p value are listed in the top right of each graph. MiRNAs most correlated with platelets are shown in the left column. MiRNAs somewhat correlated with platelets are shown in the center column. MiRNAs not significantly correlated with platelets are shown in the right column. (TIF) [file pone.0064795.s001.tif]

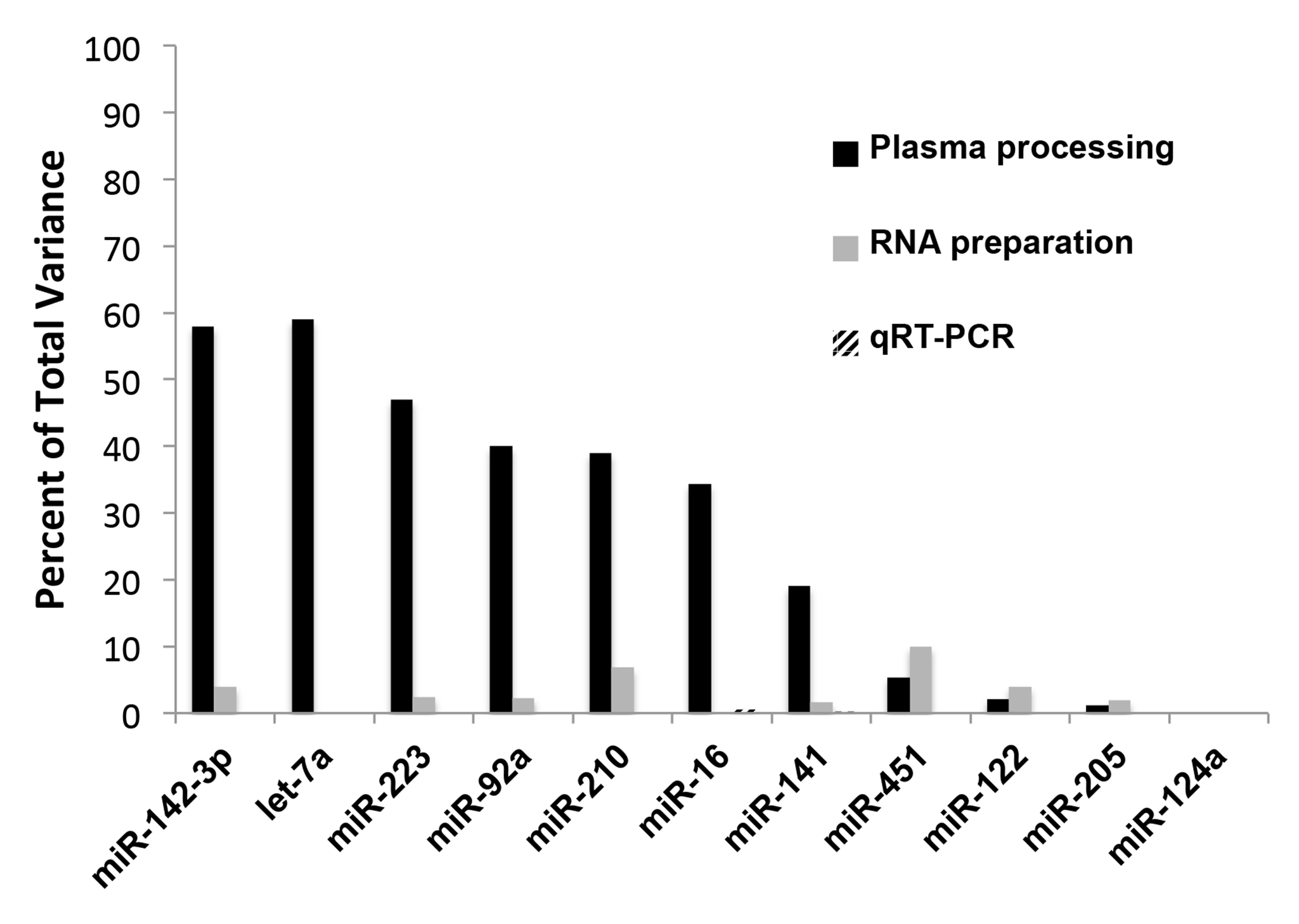

Supplement: Figure S2 — Percentage Contribution of Total Variance in miRNA Expression from Processing and Analysis Steps in Archival Blood Specimens. Contribution of total variance in miRNA expression from plasma processing, RNA preparation, qRT-PCR in the frozen archived plasma samples. Analysis of estimating components of variances to measure variation from pre-analytic (study subjects) and analytic factors (specimen type and RNA preparation) using the archived samples was conducted. This approach was based on the concept that total variance of the expression of miRNA was equal to the sum of the variances of the components [1], [2]. miR-205, miR-124a, miR-141, and miR-122 were excluded due to missing data (52%, 95%, 39%, and 16%, respectively). Among the remaining seven miRNAs, total variance ranged from 1.46 (miR-16) to 5.42 (miR-142-3p). Inter-assay variance contributed to most of the total variance (38%–85%). Intra-assay variance was ranged from 0.33 (miR-451) to 3.36 (miR-143-3p) among the seven miRNAs. Within intra-assay factors (centrifugation, RNA preparation, and duplicated PCR), centrifugation contributed more than 50% among eight of eleven miRNAs and the range was from 91.1% to 100.0%. (Not shown is the contribution of biological variance, which makes up the remaining difference.) 1. McDonald JS, Milosevic D, Reddi HV, Grebe SK, Algeciras-Schimnich A (2011) Analysis of circulating microRNA: preanalytical and analytical challenges. Clin Chem 57∶833–840. 2. Tichopad A, Kitchen R, Riedmaier I, Becker C, Stahlberg A, et al. (2009) Design and optimization of reverse-transcription quantitative PCR experiments. Clin Chem 55∶1816–1823. (TIF) [file pone.0064795.s002.tif]
